# The Masquerades of a Childhood Ciliary Body Medulloepithelioma: A Case of Chronic Uveitis, Cataract, and Secondary Glaucoma

**DOI:** 10.1155/2012/493493

**Published:** 2012-04-04

**Authors:** Jocelyn Chua, Wisam J. Muen, Ashwin Reddy, John Brookes

**Affiliations:** ^1^Moorfields Eye Hospital, City Road, London EC1V 2PD, UK; ^2^Glaucoma Department, Singapore National Eye Centre, 11 Third Hospital Avenue, Singapore 168751; ^3^Retinoblastoma Service, Royal London Hospital Barts and The London NHS Trust, Whitechapel Road, London E1 1BB, UK; ^4^Addenbrooke's Hospital, Cambridge University Hospitals, Hills Road, Cambridge CB2 0QQ, UK

## Abstract

Ciliary body medulloepitheliomas in childhood often masquerade other intraocular conditions due to its insidious nature as well as its secondary effects on proximal intraocular tissues in the anterior chamber. We report a case where a ciliary body medulloepithelioma in a two-year-old boy presents with chronic uveitis, cataract, and an uncontrolled secondary glaucoma after an innocuous blunt ocular trauma. The diagnosis was only made after the occurrence of a ciliary body mass. We discuss the clinical features of ciliary body medulloepitheliomas, the implications of a delayed diagnosis and treatment as well as the concern of periorbital tumor seeding with the use of an aqueous shunt implant in this case.

## 1. Introduction

The diagnosis of a ciliary body medulloepithelioma is usually promptly made in the presence of a ciliary body mass. However, when the tumor masquerades as other intraocular conditions, diagnosis is often delayed. We describe such a case in a two-year-old child.

## 2. Case Presentation

A healthy two-year-old white boy sustained a blunt injury to his right eye after hitting against a sideboard in September 2008. He was asymptomatic till a month later when he presented to a local Casualty Department with a red and painful right eye. Upon referral to a children's hospital, he had light perception vision in his right eye and strongly objected to occlusion of his fellow eye. Examination of his right eye under anesthesia revealed conjunctival injection, corneal stromal haze, an intense anterior chamber reaction with fibrinous reaction and iris deposits, lens opacity as well as a raised intraocular pressure of 30 mmHg. The corneal diameters were 12 mm bilaterally. Examination of the posterior segment was difficult due to significant media opacity but the retina appeared attached. Examination of his left eye was unremarkable. He was otherwise systemically well without any previous medical or ophthalmic history.

An anterior chamber tap was performed and this revealed small round cells in clumps. As the specimen was poorly preserved, no further evaluation was possible. There was no mass lesion seen on a magnetic resonance imaging scan of the right orbit. An ultrasound scan showed clumps of hyperechogenic material over the temporal lens surface, which was found to be adjacent to but not apposed to the ciliary body and extruding into the anterior vitreous. This was associated with a cataractous lens and the presence of cyclitic membranes. The diagnosis of a traumatic cataract with severe uveitis of the right eye was made and intensive topical steroid therapy was started to treat the inflammation. He later underwent a right lensectomy and anterior vitrectomy on January 2009 (four months after initial presentation) and was left aphakic.

Postoperatively, the intraocular pressure in his right eye became increasingly difficult to control despite the use of gutt Cosopt and Travatan. The cup-disc ratio in his right eye was then noted to be 0.5 (compared to 0.1 in the left) and the right axial length 23.6 mm (compared to 21.2 mm in the left). The angle was closed in all 4 quadrants and an ectropion uvea was observed. There was no rubeosis. A limited trans-scleral cyclodiode laser was performed to the right eye two weeks later. He then underwent a right Baerveldt shunt (350 mm^2^) with 0.4 mg/mL Mitomycin-C in September 2009 due to further glaucoma progression, as evident by an increase in the cup-disc ratio (0.7), axial length (25.8 mm), corneal diameter (14 mm), and intraocular pressure measurement (39 mmHg).

**Figure 1:**
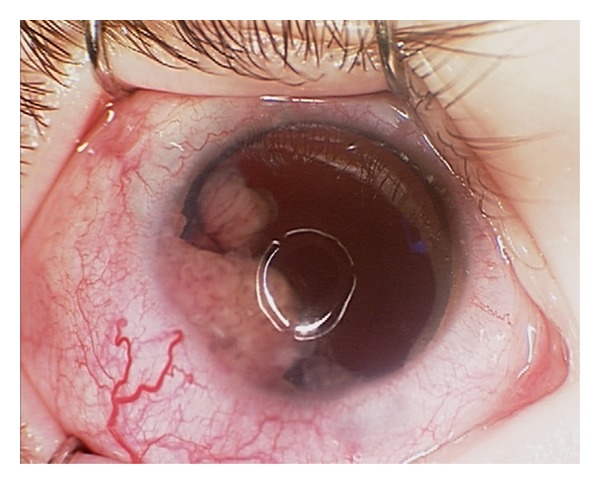
A solid flesh-coloured fibrovascular mass lesion was seen in the inferotemporal part of the child's right eye. This was associated with conjunctival injection, sentinel episcleral vessel, and anterior uveitis as well as an ectropion uvea. The right eye was aphakic.

**Figure 2:**
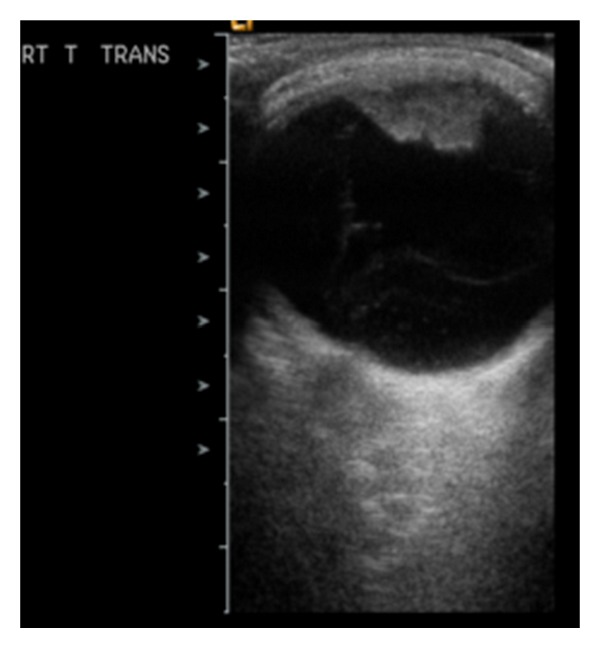
An irregular-surfaced ciliary body mass with heterogeneous echogenicity as seen on B-scan ultrasound imaging. There was no satellite lesion.

In February 2010 (about 15 months after initial presentation), his parents observed a rapidly enlarging mass lesion in the inferotemporal part of the child's right eye over a week's duration. On examination of his right eye, a solid flesh-coloured fibrovascular inferotemporal ciliary body mass was seen in the presence of sentinel vessels (Figure 1) and this was associated with significant vitreous seeding. An iridocorneal touch at the 7 o'clock position and the presence of an ectropion uvea were observed. His right vision remained light perception and intraocular pressure was controlled at 12 mmHg. Ultrasound imaging revealed an irregular-surfaced mass with heterogeneous echogenicity measuring 8 mm in length by 11.3 mm in width by 5.4 mm in thickness (Figure 2). No satellite mass was seen. A diagnosis of ciliary body medulloepithelioma was made based on the clinical appearance and location of the lesion as well as its ultrasonographic features. Prompt enucleation of the right eye was then performed in view of a possible malignant transformation due to its rapid clinical growth as well as an underlying poor visual prognosis. Histology revealed a locally invasive anterior ciliary body tumor comprising of undifferentiated highly mitotic cells and in keeping with the clinical diagnosis of a ciliary body medulloepithelioma. No rosette was seen. Due to the histologically malignant features as well as a previous Baerveldt tube implant (increased risk of periorbital tumor seeding), he was further treated with six cycles of chemotherapy using vincristine, etoposide, and carboplatin. No recurrence was observed after 14 months of followup.

## 3. Discussion

This case highlights the extent at which a diagnosis was delayed, when a ciliary body medulloepithelioma in a two-year-old child masquerades as a chronic uveitis, cataract, and secondary glaucoma. The history of an ipsilateral blunt ocular trauma was initially assumed to be responsible for the diagnoses of a traumatic uveitis and cataract as well as a subsequent secondary inflammatory glaucoma.

Ciliary body medulloepithelioma is the commonest ciliary body neoplasm in childhood. Grinker coined this term “medulloepithelioma” in 1931 as it best describes the cellular derivation of this neoplasm from the undifferentiated medullary epithelium of the embryonic retinal epithelium destined to form the nonpigmented ciliary body epithelium during the later years of life. It is most commonly found at the ciliary body. It can be benign or malignant like in this case and may exhibit teratoid features.

Several clinical and demographic features, characteristic of ciliary body medulloepitheliomas, were present in our case [1, 2]. These tumors are known to occur sporadically during the first decade of life as unilateral, solitary amelanotic flesh-coloured solid lobulated lesions arising from the ciliary body. Ultrasonographic features of an irregular-surfaced lesion with cystic cavities and irregular internal reflectivity on A-scan further supported this clinical diagnosis [2, 3]. Unfortunately, these clinical features were observed late. Previous case reports [2, 4–6] have shown that diagnosis is usually unequivocal when a ciliary body mass presents in childhood. However, other differential diagnoses of a ciliary body mass in childhood, such as retinoblastoma, malignant melanoma, juvenile xanthogranuloma, or ocular metastatic lesion, should also be considered. Due to the proximity of the tumor to adjacent intraocular structures as well as its secondary effects, complications [7] such as cataract, ectopia lentis, secondary angle closure, rubeotic glaucoma, uveitis, ectropion uvea, or vitreous hemorrhage may present first before a ciliary body mass becomes clinically apparent. Broughton and Zimmerman [8] reviewed 56 cases and found that up to 20% of cases were misdiagnosed and surgically treated for other conditions with procedures such as lensectomy or glaucoma drainage procedures and the diagnosis delayed for up to one year. Given the prior history of a blunt ocular injury in our case, the initial diagnosis of a traumatic cataract and uveitis was certainly plausible. However, our preoperative observations of iris deposits and cyclitic membranes, clinical features which were similar to earlier case reports [9, 10], might have been salient clues to suggest an underlying neoplastic process. The ensuing secondary angle closure glaucoma was then attributed to several factors such as trauma, uveitis as well as an aphakic status. Insertion of an aqueous shunt was performed to relieve the intraocular pressure but in retrospect, it might have led to an inadvertent tumor seeding into the periorbital space. Should the clinical diagnosis of a neoplasm been made earlier, a filtration surgery would not have been the procedure of choice.

Surgical removal of the tumor provides a definitive histological diagnosis. The decision for enucleation is usually based on a large tumor size, a painful eye with poor visual potential, and a strong clinical suspicion of malignancy for instance rapid tumor growth as in our case. As ciliary body medulloepitheliomas are generally locally invasive, complete excision is curative and associated with a good survival prognosis. In our case, the delayed diagnosis might have contributed to the local scleral invasion of the tumor, thereby necessitating adjuvant chemotherapy after enucleation.

In conclusion, the differential diagnosis of an intraocular neoplasm should be considered when a child presents with a severe chronic uveitis associated with cyclitic membranes, and an ultrasound biomicroscopy should be carried out to exclude a ciliary body neoplasm.
